# Understanding anhedonia in major depressive disorder in Japan: epidemiology and unmet needs from patients’ and physicians’ perspectives

**DOI:** 10.1186/s12888-025-07089-4

**Published:** 2025-07-01

**Authors:** Mami Kasahara-Kiritani, Tadafumi Kato, Akihide Wakamatsu, Thomas Webb, Keira Herr, Lawrence Vandervoort, Nan Li

**Affiliations:** 1 Integrated Market Access Division, Johnson & Johnson, Tokyo, Japan; 2 Department of Psychiatry and Behavioral Science, Juntendo University Graduate School of Medicine, Tokyo, Japan; 3 Medical Affairs Division, Johnson & Johnson, Tokyo, Japan; 4 Johnson & Johnson, Singapore, Singapore; 5 Oracle Life Sciences, Singapore, Singapore; 6 3-5-2 Nishi-Kanda, Chiyoda-ku, Tokyo, 101-0065 Japan

**Keywords:** Anhedonia, Japan, Major depressive disorder, Physicians' perspectives, Prevalence, Treatment goals, Treatment satisfaction

## Abstract

**Background:**

Anhedonia (ANH), one of the core symptoms of major depressive disorder (MDD), poses a significant health challenge. We evaluated the prevalence of ANH among MDD patients in Japan, and elucidated patient journey from patients’ and physicians’ perspective.

**Methods:**

This cross-sectional observational study (April-May 2023) utilized a self-reported, online-based survey targeting the general population (non-physicians) and physicians. The general population (aged ≥ 18 years) were screened for MDD using Patient Health Questionnaire-9 (PHQ-9 ≥ 10); MDD patients were further screened for ANH using Snaith-Hamilton Pleasure Scale (SHAPS; MDD-ANH: SHAPS > 2, MDD non-ANH: SHAPS ≤ 2). The age- and gender-weighted prevalences of MDD and MDD-ANH, patient journey, and treatment goals and satisfaction between patients and physicians were reported. P-value < 0.05 was considered statistically significant.

**Results:**

The prevalence of MDD was 3.4% (*n* = 514; *N* = 15,266) and the prevalence of ANH in MDD was 66.9% (*n* = 344). Mean (± standard deviation) age of MDD-ANH patients (*n* = 282) was 46.1 ± 12.5 years, while for MDD non-ANH patients (*n* = 50) was 49.6 ± 8.5 years. Physicians (*n* = 60) had mean 21.9 years of experience working as psychiatrists. Physicians reported that 33.9% of their MDD patients had anhedonia. MDD-ANH patients scored significantly higher (*p* < 0.05) than MDD non-ANH patients on all PHQ-9 items, except for feeling tired/having little energy and poor appetite/overeating. A higher percentage of MDD-ANH patients reported current prescription use for depression than MDD non-ANH patients (67.0% vs. 51.3%; *p* = 0.0677). Treatment duration with multiple prescriptions was significantly longer in MDD-ANH than MDD non-ANH patients (102.1 ± 89.8 vs. 53.8 ± 33.7 months; *p* = 0.0035). The majority of physicians (90.0%) reported that they do not focus on treating anhedonia separately from MDD. Patients with MDD-ANH perceived “reduce psychological anxiety”, “control depressed mood”, and “improve sleep quality” as more important treatment goals, compared to physicians’ importance to avoid suicidal thoughts, restore normal social function, and regain interest in hobbies. Treatment satisfaction levels were higher among physicians than MDD-ANH patients across all treatment goals.

**Conclusion:**

This study in Japan reported high prevalence of ANH among MDD patients which was significantly underestimated by physicians. Discordances in treatment goals and satisfaction were observed between physicians and MDD patients, highlighting the need for aligning patient and physician expectations.

**Trial registration:**

Not applicable.

**Supplementary Information:**

The online version contains supplementary material available at 10.1186/s12888-025-07089-4.

## Introduction

In the landscape of mental health disorders, major depressive disorder (MDD) stands as a leading cause of disability-adjusted life years (DALYs), wielding a substantial impact on public health globally (affecting an estimated 246 million individuals) [1–3]. A nationwide survey conducted in Japan (World Mental Health Japan 2nd ) from 2013 to 2015 found that 5.7% of the population experienced lifetime prevalence of MDD, with a 12-month prevalence of 2.7% [4]. Moreover, Japan faces considerable challenges due to its relatively high suicide rates (ranking ninth globally), underscoring the critical need to address MDD and its associated symptoms within this demographic [5]. 

Anhedonia, one of the core symptoms of various mental health disorders, poses a significant challenge within the fields of psychiatry and public health [1, 6–8]. According to the fifth edition of The Diagnostic and Statistical Manual of Mental Disorders, Text Revision (DSM-5-TR), anhedonia is a key symptom required for diagnosing major depressive episodes [9]. Anhedonia is defined as “lack of enjoyment from, engagement in, or energy for life’s experiences; deficits in the capacity to feel pleasure and take interest in things” [9]. Existing literature indicates that anhedonia prevalence varies across mental health disorders - approximately 35–70% in MDD [10–12], 52% in bipolar depression [13], and around 40–80% in schizophrenia [14]. 

Current literature explains depression as a syndrome rather than a single disorder, with a heterogeneous presentation of emotional, physical, and cognitive symptoms having significant impact on patients’ functioning and health-related quality of life [15, 16]. Mechanism of anhedonia, an aspect of emotional blunting in patients with depression, has been linked to dysfunctions in fronto-striatal and prefrontal dopaminergic pathways [8, 15, 17]. Anhedonia is implicated in impeding treatment-related outcomes in various mental health disorders, and often proves resistant to first-line antidepressant treatments [8, 18]. Moreover, anhedonia may persist in patients with MDD following subsequent treatment strategies such as augmentation or switching, and continue to affect quality of life, making anhedonia as an important treatment target in the wider treatment of MDD [19]. Adding to the complexity is the lack of treatment guidelines for anhedonia [20, 21]. MDD patients who experience anhedonia might often endure extended treatment durations and potentially take multiple medications, as conventional therapies fail to yield sufficient effectiveness in alleviating this debilitating symptom [1, 8]. Consequently, the administration of multiple psychotropic medications may potentially emerge as a common practice, emphasizing the challenge in effectively managing anhedonia in MDD.

Despite its prevalence, anhedonia remains relatively understudied in Asia, specifically in Japan, concerning its specific impact on MDD. Existing research on anhedonia in Japan has primarily centered on its association with Parkinson’s Disease [22–25] and postnatal depression in women [26],, leaving a notable gap in understanding its implications within the context of MDD. This limited focus suggests a lack of approved interventions targeting anhedonia in the clinical setting for MDD [27, 28]. Understanding the patient journey of MDD patients experiencing anhedonia is crucial to mitigate the negative impact of anhedonia on MDD outcomes in Japan.

Therefore, the current study aimed to evaluate the prevalence of anhedonia among patients with MDD in Japan. To support a better understanding of MDD patients with traits of anhedonia in Japan, this study also aimed to elucidate the patient journey from both patient and physicians’ perspective. Furthermore, this study investigated patient and physician concordance/discordance of the importance of treatment goals and the satisfaction with current pharmaceutical treatments in achieving those treatment goals.

## Methods

### Study design

This cross-sectional observational study which was conducted during April-May 2023 collected real-world data in Japan with a self-reported, online-based survey. The study comprised two surveys: one targeting the general population (non-physicians), and another survey administered to physicians. The general population-specific survey aimed to assess the prevalence of MDD and anhedonia in Japan, as well as patients’ characteristics including sociodemographic and general health information, also to understand the patient journey among patients with MDD and anhedonia, and their treatment goals and satisfaction related to managing anhedonia. The physician-specific survey was designed to assess physicians’ characteristics, such as clinical experience and patient load, physicians’ perspectives on anhedonia, treatment goals, and treatment satisfaction to manage anhedonia in patients with MDD.

### Data source

**Fig. 1:**
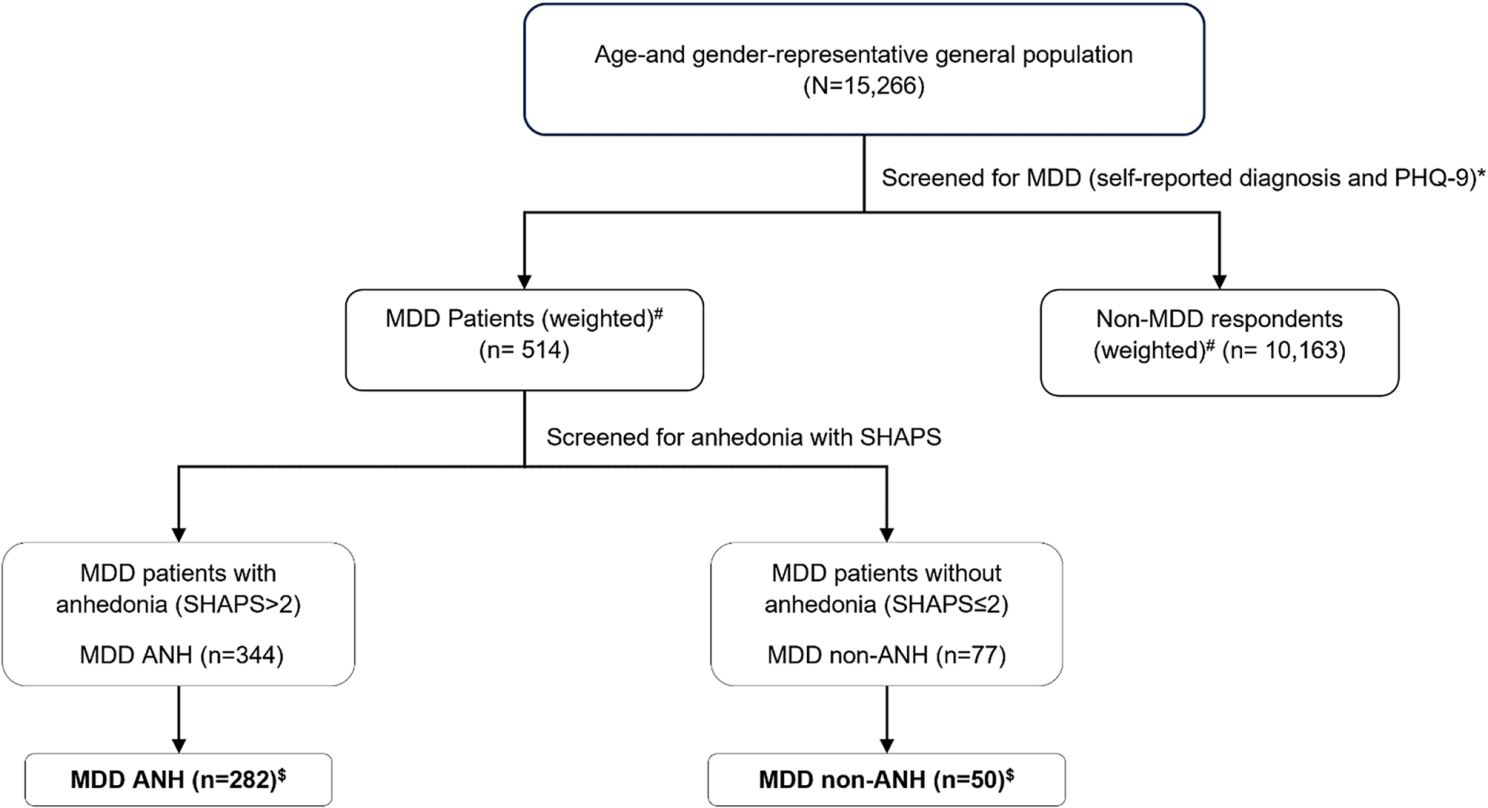
Study design. * MDD without diagnosis of bipolar disorder or schizophrenia. ^#^ MDD patients with self-reported diagnosis and PHQ-9 score ≥ 10; *n* = 93 did not complete the SHAPS questionnaire. ^$^MDD Patients who completed all survey questions and included in analysis

The general population (non-physician) respondents were recruited through opt-in databases designed to reflect the gender and age group distribution of the most recent nationwide census in Japan. Each respondent underwent a screening process to ensure they met the study criteria; those eligible provided electronic consent and entered the 20–30-minute survey. These respondents were screened for MDD and those with MDD were further screened for anhedonia (Fig. 1). The respondents received a nominal incentive upon completion of the survey. Physicians were recruited from the Medical Database Provision Business (MDB) in Japan using purposive sampling. Physicians completed the screener, those eligible provided electronic consent and were invited to complete a 15-minute survey. Physicians received an honorarium after completion of the web-based survey. No further follow-ups or monitoring were conducted on either general population respondents or physicians after the completion of the survey.

The study adhered to the ethical principles outlined in the Declaration of Helsinki and followed Good Epidemiological Practices (GEP) as defined by the International Society for Pharmacoeconomics and Outcomes Research (ISPOR). Approval for the study protocol was obtained from the Toukeikai Kitamachi Clinic Ethical Review Board in Japan (approval number: EJP09413). Appropriate confidentiality measures were implemented throughout the study to safeguard the privacy of all respondents’ records, ensuring that individual respondents always remain unidentified.

### Study population

#### General population (non-physicians)

Respondents aged ≥ 18 years, residing in Japan without self-reported diagnosis of bipolar disorder or schizophrenia, who were non-physicians were recruited. MDD patients were defined as respondents who self-reported both physician diagnosis of MDD and the presence of MDD symptoms within the past 2 weeks, as indicated by the Patient Health Questionnaire-9 (PHQ-9) score of ≥ 10 [29, 30]. MDD patients were further stratified into two groups; MDD-ANH, and MDD non-ANH based on Snaith-Hamilton Pleasure Scale (SHAPS) score. SHAPS is a validated instrument designed to assess pleasure response [31]. The SHAPS score is a self-reported questionnaire comprising of 14 items that measures inability to experience pleasure across domains such as social activities, sensory stimuli (eating tasty food and drink), hobbies and pastimes [31, 32]. The questions are rated on a 2-point Likert scale (1 = strongly disagree, 1 = disagree, 0 = agree, 0 = strongly agree). The total SHAPS score ranges from 0 to 14, with a score of ≤ 2 being considered normal [31]. In the current study, MDD respondents with SHAPS score > 2 were categorized as MDD-ANH, while MDD respondents with SHAPS score of ≤ 2 were categorized as MDD non-ANH [33].

#### Physicians

Physicians eligible for participation in the physician-specific survey were required to be practicing psychiatrists with a minimum of three years of clinical experience, spent at least 30% of their time in direct patient care, and treated a minimum of 20 MDD patients in the last month.

### Study measures

#### Prevalence of MDD and MDD with anhedonia

This study is a part of the regional study; however, the present study has focused on reporting the findings of the Japanese population only. The prevalence of MDD was determined by dividing the total number of respondents who self-reported an MDD diagnosis and had a PHQ-9 score of ≥ 10 [30] by the total number of eligible respondents in the study. The prevalence of MDD and anhedonia was calculated by dividing the number of respondents with MDD who, had SHAPS score > 2 [33] by the total number of respondents with MDD. To mitigate potential sampling biases and ensure greater representativeness to the national population, prevalence rates were age- and gender-weighted by using 2022 United Nations population estimates for Japan [34]. 

#### Patient and physician characteristics

This study assessed the characteristics (weighted) of patients with MDD-ANH and MDD non-ANH, including their sociodemographic (sex, age, education, and employment status) and general health characteristics (body mass index [BMI], smoking frequency, frequency of alcohol consumption, and Charlson Comorbidity Index [CCI; a higher total index score is an indicator of severe comorbidities]) [35]. 

Additionally, the study assessed physicians’ characteristics (weighted) such as years practicing as a psychiatrist, percentage of time in direct patient care, number of MDD patients seen in the past month, and patient caseload (percentage breakdown of MDD severity among MDD patients, percentage of MDD-ANH patients, percentage breakdown of anhedonia severity among MDD-ANH patients).

#### Patient journey from patient’s perspective

This study measured the weighted depression-specific characteristics for MDD-ANH vs. MDD non-ANH such as time since depression diagnosis (in years), depression symptoms prompting diagnosis, diagnosing physician, PHQ-9 items, prescription use for depression, duration of treatment in months, prior medication use and reasons for switching.

#### Physicians’ perspectives on anhedonia in MDD

Physicians’ perspectives towards anhedonia based on their clinical experience and clinical patient caseload were measured by their assessment of anhedonia and overall depression severity in their clinical setting, treatment approaches for MDD-ANH patients, extent of agreement with the definition of anhedonia, and extent of agreement with statements regarding anhedonia in MDD. Physicians’ agreement to definitions of anhedonia and statements about anhedonia in MDD were assessed using exploratory statements, with responses collected on a Likert scale ranging from 1 to 9 (1 = strongly disagree to 9 = strongly agree).

#### Treatment expectations (goals) and satisfaction

Additionally, this study assessed patient and physician alignment on treatment goals and satisfaction with pharmaceutical treatments. Both MDD and physician respondents rated the level of importance of treatment goals using a Likert scale ranging from 1 to 5 (1 = not at all important, 5 = extremely important) and expressed treatment satisfaction on a Likert scale ranging from 1 to 9 (1 = extremely dissatisfied, 9 = extremely satisfied). Higher scores indicated greater importance or satisfaction with treatment.

### Statistical analysis

Data analyses included both descriptive and inferential approaches to examine responses from patient and physician surveys. All outcomes of the study were subjected to descriptive analysis. Mean values with their corresponding standard deviations (SD) were used to represent continuous variables, whereas frequencies and percentages were employed for categorical variables. The prevalence of MDD and MDD-ANH based on age- and gender-weighted PHQ-9 and SHAPS scores, characteristics (weighted) of patients with MDD-ANH and MDD non-ANH, physicians’ characteristics (weighted) and responses to the survey questions were analyzed descriptively.

Inferential analyses were performed to identify the differences between MDD-ANH and MDD non-ANH patients, utilizing age- and gender-weighted responses to the survey questions. Furthermore, comparisons were made between groups across different parameters which include depression-specific characteristics and treatment outcomes (between MDD-ANH and MDD non-ANH respondents), as well as treatment002Drelated outcomes such as treatment goals and satisfaction (between both MDD ANH vs. physicians and MDD non-ANH vs. physicians). Chi-square tests (categorical variables) and t-tests (continuous variables) were employed to evaluate differences between groups. For all significance testing, p-values < 0.05 (two-tailed) was considered statistically significant.

All statistical analyses were conducted using SPSS version 29 (IBM) and/or R 4.2.2 and/or SAS 9.4.

## Results

### Prevalence of MDD and MDD with anhedonia in Japan

Of the total survey sample (*N* = 15,266), 1944 respondents (12.7%) demonstrated a PHQ-9 score ≥ 10, indicating moderate or greater depression severity. Age- and gender-weighted prevalence of MDD was reported as 3.4% (*n* = 514) among survey respondents. Among the respondents with MDD, 43.2% (*n* = 222), 28.4% (*n* = 146) and 28.4% (*n* = 146) had moderate, moderate-severe and severe depression, respectively. The age- and gender-weighted prevalence of anhedonia among MDD patients was 66.9% (*n* = 344) (Table 1).

**Table 1:** Age and gender-weighted PHQ-9 and SHAPS among total survey and MDD samples in Japan

Variables	Survey sample	MDD sample^†^
	*N* = 15,266	*N* = 514
**PHQ-9**, ***n*(%)**		
No Depression (0–4)	10,163 (66.6)	0 (0.0)
Mild (5–9)	3,159 (20.7)	0 (0.0)
Moderate (10–14)	1,121 (7.3)	222 (43.2)
Moderate-severe (15–19)	467 (3.1)	146 (28.4)
Severe (20–27)	356 (2.3)	146 (28.4)
SHAPS^*^, **n (%)**		
0	30 (0.2)	30 (5.8)
1–2	47 (0.3)	47 (9.1)
Presence of anhedonia (≥ 3)	344 (2.3)	344 (66.9)

MDD, major depressive disorder; PHQ, Patient Health Questionnaire; SHAPS, Snaith-Hamilton Pleasure Scale

*SHAPS is surveyed among respondents with moderate depression or greater (PHQ-9 ≥ 10) and a self-reported physician diagnosis of depression

^†^ Respondents are classified under MDD if they reported moderate depression or greater (PHQ-9 ≥ 10) and a self-reported physician diagnosis of depression

Note: Prevalence weights were produced using UN population estimates for Japan

### Patients’ characteristics

#### Sociodemographic and general health characteristics

Patients with MDD-ANH (*n* = 282) were aged 46.1 ± 12.5 (mean ± SD) years, slightly lower than the MDD non-ANH patients (*n* = 50) who were aged 49.6 ± 8.5 years. The percentage of male patients in MDD-ANH group (41.8%) was numerically higher compared to MDD non-ANH group (29.7%). Majority of the patients in both MDD-ANH group (65.8%) and MDD non-ANH (71.9%) group were employed (Table 2).

**Table 2:** Patients’ characteristics (weighted)

Variables	MDD-ANH (*n* = 282)	MDD non-ANH (*n* = 50)
**Sociodemographic characteristics**		
**Sex**, ***n*(%)**		
Male	118 (41.8)	15 (29.7)
Female	164 (58.2)	35 (70.3)
**Age**,** mean (SD)**	46.1 (12.5)	49.6 (8.5)
**Age distribution**,** n (%)**		
18 to < 25	32 (11.2)	0 (0.0)
25 to < 35	27 (9.5)	2 (3.6)
35 to < 45	62 (21.8)	10 (19.3)
45 to < 55	88 (31.0)	24 (49.0)
55 to < 65	49 (17.5)	12 (23.7)
65 and older	25 (9.0)	2 (4.5)
**Education**,** n (%)**		
Elementary school	0 (0.0)	0 (0.0)
Junior high school	7 (2.5)	1 (1.2)
High school	84 (29.9)	15 (30.5)
2 year college	55 (19.4)	7 (14.4)
4 year college	96 (33.9)	17 (34.9)
Graduate school or above	21 (7.3)	6 (13.0)
No school	0 (0.0)	0 (0.0)
Others	9 (3.1)	3 (6.0)
Decline to answer	11 (4.0)	0 (0.0)
**Employment status**,** n (%)**		
Employed*	186 (65.8)	36 (71.9)
Non-employed^†^	99 (35.2)	15 (30.6)
**General health characteristics**		
**BMI**,** mean (SD)**	22.89 (5.1)	22.4 (4.2)
**BMI categories**,** n (%)**		
Underweight (< 18.5)	47 (16.7)	9 (18.1)
Normal weight (18.5 to < 25)	163 (57.7)	31 (62.9)
Overweight (25 to < 30)	46 (16.4)	6 (11.7)
Obese (30 or greater)	26 (9.2)	4 (7.3)
Unknown	0 (0.0)	0 (0.0)
**Frequency of smoking**,** n (%)**		
Every day	80 (28.2)	9 (18.9)
Some days	28 (10.0)	1 (1.2)
Not at all	175 (61.8)	40 (80.0)
**Frequency of consuming alcohol**,** n (%)**		
Every day	66 (23.4)	10 (19.4)
Some days	120 (42.4)	20 (40.7)
Not at all	97 (34.3)	20 (39.9)
**CCI**,** mean (SD)**	1.1 (1.5)	1.2 (1.5)
**CCI score categories**,** n (%)**		
0	129 (45.7)	20 (39.5)
1	66 (23.3)	17 (34.6)
2	50 (17.5)	5 (9.6)
3+	38 (13.5)	8 (16.4)

ANH, anhedonia; BMI, body mass index; CCI, Charlson Comorbidity Index; MDD, major depressive disorder; SD, standard deviation

*Employed comprises of respondents who are employed full-time or part-time and self-employed

^†^Non-employed comprises of respondents who are homemakers, retired, student, long-term disability (long-term leave of absence due to illness of your own [more than 3 months]), short-term disability (short-term leave of absence due to illness of your own [less than 3 months]), not employed but looking for work, not employed and not looking for work

In terms of general health characteristics, patients with MDD-ANH had a slightly higher mean BMI (22.9 ± 5.1 vs. 22.4 ± 4.2) and a higher percentage of patients with MDD-ANH reported smoking every day (28.2% vs. 18.9%) than MDD non-ANH patients. Similarly, patients with MDD-ANH reported higher rates of alcohol consumption every day (23.4%) compared to MDD non-ANH patients (19.4%). The mean CCI scores were slightly lower in the MDD-ANH group (1.1 ± 1.5) than the MDD non-ANH group (1.2 ± 1.5) (Table 2). The unweighted characteristics of MDD patients are presented in Table S1.

#### Physicians

In this study, 60 eligible physicians were recruited and completed the survey. The physicians reported having a mean experience of 21.9±9.0 years practicing as psychiatrists and allocated an average of 88.0% of their time to direct patient care. In terms of patient caseload, physicians reported seeing an average of 96.4±94.5 MDD patients in the past month. Physicians reported that, on average, 33.9% of their patients with MDD had anhedonia. Among MDD-ANH patients, physicians observed varying degrees of severity, with 48.0%, 34.6%, and 17.5% experiencing mild, moderate, and severe anhedonia, respectively (Table 3).

**Table 3:** Demographics and patient caseload for physicians

Variables	Physicians (*N* = 60)
Years practicing as a psychiatrist, mean (SD)	21.9 (9.0)
Percentage of time in direct patient care, mean (SD)	88.0 (9.8)
Number of MDD patients seen in the past month, mean (SD)	96.4 (94.5)
**Patient caseload**	
**Percentage breakdown of MDD severity among MDD patients**,** mean (SD)**	
% of mild MDD patients	45.9 (19.1)
% of moderate MDD patients	39.1 (13.6)
% of severe MDD patients	15.0 (11.3)
**Percentage of MDD patients with ANH**,** mean (SD)**	33.9 (20.3)
**Percentage breakdown of ANH severity among MDD patients with ANH**,** mean (SD)**	
% of MDD patients with mild ANH	*n* = 49
	48.0 (19.9)
% of MDD patients with moderate ANH	*n* = 49
	34.6 (13.4)
% of MDD patients with severe ANH	*n* = 49
	17.5 (18.2)
I don’t know the breakdown	*n* = 60
	0.2 (0.4)

ANH, anhedonia; MDD, major depressive disorder; SD, standard deviation

### Patient journey from patient’s perspective (age- and gender-weighted responses: MDD-ANH vs. MDD non-ANH)

#### Depression-related characteristics

No significant difference was observed in the time since depression diagnosis between the two groups, with MDD-ANH patients reporting a mean of 11.9 ± 8.3 years compared to 13.0 ± 7.1 years in MDD non-ANH patients (*p* = 0.3557). Additionally, both groups exhibited similar patterns in the symptoms prompting patients to seek medical consultation, with the three most common symptoms being depressed mood and other emotional problems (89.2% vs. 88.9%), sleep pattern changes (57.2% vs. 52.1%), and mental changes (40.7% vs. 40.6%) (Table 4).

**Table 4:** Depression-specific characteristics and treatment outcomes (weighted) for MDD-ANH vs. MDD non-ANH in Japan

Variables	MDD-ANH (*n* = 282)	MDD non-ANH (*n* = 50)	*p*-value
Time since depression diagnosis (years)*,** mean (SD)**	11.9 (8.3)	13.0 (7.1)	0.3557
Depression symptoms prompting diagnosis*,** n (%)**			
Depressed mood and other emotional problems (e.g. hopelessness, tiredness, anxiety)	252 (89.2)	44 (88.9)	0.9565
Eating pattern changes (e.g. appetite loss, weight loss, overeating, weight gain)	63 (22.4)	14 (27.9)	0.4194
Sleep pattern changes (e.g. difficulty sleeping, oversleeping, waking up early)	161 (57.2)	26 (52.1)	0.5414
Mental changes (e.g. forgetfulness, difficulty thinking, difficulty concentrating)	115 (40.7)	20 (40.6)	0.9955
Social problems (e.g. isolation, alcohol problems, drug problems, sex problems)	29 (10.4)	3 (6.9)	0.3445
Physical problems (e.g. headaches, body aches, constipation)	80 (28.4)	16 (31.2)	0.7154
None of these	4 (1.5)	1 (2.4)	0.6560
Diagnosing physician*,** n (%)**			
General practitioner/family practitioner	9 (3.3)	2 (4.9)	0.7689
General internist / internal medicine physician	18 (6.2)	5 (9.9)	
Psychiatrist	247 (87.5)	42 (83.7)	
Psychologist	2 (0.8)	0 (0.0)	
Other	6 (2.2)	1 (1.5)	
**PHQ-9 items**,** mean (SD)**			
Item 1 - Little interest or pleasure in doing things	1.9 (0.8)	1.3 (0.8)	**< 0.0001**
Item 2 - Feeling down, depressed, or hopeless	1.9 (0.8)	1.6 (0.9)	**0.0069**
Item 3 - Trouble falling or staying asleep, or sleeping too much	2.2 (0.8)	1.6 (0.9)	**< 0.0001**
Item 4 - Feeling tired or having little energy	2.3 (0.7)	2.4 (0.7)	0.7866
Item 5 - Poor appetite or overeating	1.8 (0.9)	1.5 (1.0)	0.1313
Item 6 - Feeling bad about yourself — or that you are a failure or have let yourself or your family down	2.1 (0.9)	1.7 (0.9)	**0.0021**
Item 7 - Trouble concentrating on things, such as reading the newspaper or watching television	1.5 (0.9)	1.0 (0.9)	**0.0008**
Item 8 - Moving or speaking so slowly that other people could have noticed? Or the opposite — being so fidgety or restless that you have been moving around a lot more than usual	1.3 (1.0)	0.9 (0.8)	**0.0229**
Item 9 - Thoughts that you would be better off dead or of hurting yourself in some way	1.6 (1.1)	0.9 (0.8)	**< 0.0001**
**Prescription use**			
Current prescription use for depression, n (%)	189 (67.0)	25 (51.3)	0.0677
Current use of multiple prescriptions for depression^†^, n (%)	80 (42.3)	9 (34.8)	0.4885
Number of current prescription medications^†^, mean (SD)	1.6 (0.8)	1.4 (0.6)	0.0739
**Duration of treatment in months**,** mean (SD)**			
Multiple prescriptions for depression^‡^	102.1 (89.8)	53.8 (33.7)	**0.0035**
**Prior medication use and reasons for switching**,** n (%)**			
Prior depression medication use*	80 (42.3)	11 (44.0)	0.8694
**Current medication replaced or added to previous medication**			
Replaced my existing medication, treatment or therapy	46 (57.8)	8 (68.4)	0.2379
Added on to my existing medication, treatment or therapy	25 (31.0)	1 (10.2)	
Not sure	9 (11.3)	2 (21.5)	
**Reasons for switching**			
Doctor’s recommendation	31 (67.4)	5 (69.4)	0.9110
To reduce side effects	8 (16.5)	1 (14.9)	0.8917
Lower cost	4 (7.7)	1 (7.4)	0.9754
I was not responding to the previous treatment	23 (49.6)	1 (15.7)	0.1006
The dosing of the current treatment is more convenient	1 (1.2)	1 (15.7)	0.0151
The form or mode of administration is more convenient	1 (1.2)	0 (0.0)	0.9300
Others	4 (7.7)	0 (0.0)	0.0725

ANH, anhedonia; MDD, major depressive disorder; PHQ, Patient Health Questionnaire; SD, standard deviation

*Asked of those reporting a depression diagnosis who answered all questions in the depression module (MDD-ANH, MDD non-ANH)

^†^Asked of those reporting having experienced depression in the past 12 months who are taking a prescription medication (MDD-ANH, MDD non-ANH)

^‡^In cases where different providers were prescribing the medications within a group, the following hierarchy was used: psychiatrist > general practitioner > general internist/internal medicine physician > other

NOTE: Age and sex prevalence weights were produced using UN population estimates for Japan

Irrespective of anhedonia status, the majority of patients with MDD (with a self-reported physician diagnosis and PHQ-9 score ≥ 10) were diagnosed by psychiatrists (MDD-ANH: 87.5% and MDD non-ANH: 83.7%). Analysis of the PHQ-9 items revealed that patients with MDD-ANH scored significantly higher (*p* < 0.05) on all individual items compared to MDD non-ANH patients, except for items #4 (feeling tired or having little energy) and #5 (poor appetite or overeating) (Table 4).

#### Treatment landscape among patients

Regarding current treatment, a higher percentage of patients with MDD-ANH reported current prescription use for depression compared to MDD non-ANH patients (67.0% vs. 51.3%), although this difference was not statistically significant (*p* = 0.0677). Among those patients using prescription medication, 42.3% of patients with MDD-ANH and 34.8% of MDD non-ANH patients reported using multiple prescriptions. The mean number of prescriptions was higher among patients with MDD-ANH (1.6 ± 0.8) than MDD non-ANH patients (1.4 ± 0.6), although this difference was not statistically significant (*p* = 0.0739). The mean duration of treatment with multiple prescriptions was significantly longer among patients with MDD-ANH compared to MDD non-ANH patients (102.1 ± 89.8 months vs. 53.8 ± 33.7 months; *p* = 0.0035) (Table 4). Further, the percentage of patients who reported prior medication use for depression was similar between MDD-ANH and MDD non-ANH groups (42.3% vs. 44.0%). No statistically significant difference was observed between MDD-ANH and MDD non-ANH groups in the percentage of patients who had switched (57.8% vs. 68.4%) or augmented (31.0% vs. 10.2%; overall *p* = 0.2379) their medication. In terms of reasons for switching, a higher percentage of patients with MDD, regardless of anhedonia status, reported switching medication based on their doctor’s recommendations (MDD-ANH: 67.4%, MDD non-ANH: 69.4%) compared to reasons such as not responding to their previous treatment (MDD-ANH: 49.6%, MDD non-ANH: 15.7%) or to reduce side effects (MDD-ANH: 16.5%, MDD non-ANH: 14.9%) (Table 4).

### Physicians’ perspectives on anhedonia in MDD

The majority (90.0%) of physicians reported that they do not focus on treating anhedonia separately from MDD. Regarding the extent of agreement with statements related to anhedonia, physicians generally displayed a moderate level of agreement. On average, physicians reported a relatively high level of agreement with the definition of anhedonia in MDD (7.4 ± 1.0). However, most statements related to anhedonia were scored below 7 out of 9, where 9 indicates “strongly agree.” Particularly, four statements received ratings ≤ 6: physicians found it challenging to diagnose anhedonia in MDD patients in clinical practice (5.5 ± 1.9), believed that MDD-ANH patients require different treatments compared to MDD non-ANH patients (5.7 ± 1.5), perceived clinical value and importance in defining MDD-ANH as a subtype of MDD (5.8 ± 1.6), and acknowledged an inconsistent conceptualization and a lack of both an accurate and consistent definition of anhedonia in MDD (6.0 ± 1.4) (Table 5).

**Table 5:** Physicians’ perspectives on anhedonia in MDD

Variables	Physicians (*N* = 60)
**When treating MDD-ANH patients**, ***n*(%)**	
I focus on treating anhedonia separately from non-anhedonic symptoms for my MDD-ANH patients	6 (10.0)
I do not focus on treating anhedonia separately from non-anhedonic symptoms for my MDD-ANH patients	54 (90.0))
**Extent of agreement with the definition of anhedonia in MDD**,** mean (SD)**	7.4 (1.0)
**Extent of agreement with statements about anhedonia in MDD (Range 1 [strongly disagree] to 9 [strongly agree])**,** mean (SD)**	
Currently, there is an inconsistent conceptualization and therefore, a lack of both an accurate and consistent definition of anhedonia in MDD	6.0 (1.4)
Overall, there is clinical value and importance in defining MDD-ANH as a subtype of MDD	5.8 (1.6)
Anhedonia is a core symptom of MDD	6.4 (1.5)
It is difficult to diagnose anhedonia in MDD patients in clinical practice	5.5 (1.9)
It is difficult to treat anhedonia in MDD patients in clinical practice	6.1 (1.7)
It is important to diagnose anhedonia in my MDD patients	6.7 (1.3)
It is important to treat anhedonia in my MDD patients	7.2 (1.1)
MDD-ANH patients require different treatments vs. MDD non-ANH patients	5.7 (1.5)
When pharmaceutical treatments improve symptoms in MDD-ANH patients, their anhedonic and non-anhedonic symptoms improve alongside each other	6.2 (1.5)
Anhedonia is very similar to melancholia	6.6 (1.3)

MDD, major depressive disorder; MDD-ANH, MDD with anhedonia; MDD non-ANH, MDD without anhedonia; SD, standard deviation

### Treatment expectations (goals) and satisfaction towards treatment for anhedonia in MDD

The perceived level of importance of treatment goals (on scale of 1–5 with 5 being “extremely important”) varied among patients with MDD-ANH and MDD non-ANH, and physicians. Among patients with MDD-ANH, the top three treatment goals considered more important were reducing psychological anxiety (3.9 ± 1.0), improving sleep quality (3.8 ± 1.0), and controlling depressed mood (3.8 ± 1.0). Similarly, patients with MDD non-ANH prioritized reducing psychological anxiety (4.0 ± 1.2), improving sleep quality (3.9 ± 1.2), and having positive emotions (3.8 ± 1.1). In contrast, physicians emphasized the importance of restoring normal social function (3.9 ± 0.8), avoiding suicidal thoughts (3.9 ± 0.9), and regaining interest in hobbies (3.8 ± 0.8). Notably, significant differences were observed between patients with MDD-ANH and physicians in treatment expectations, particularly regarding goals related to regaining appetite (2.6 ± 1.1 vs. 3.6 ± 0.8), regaining interest in hobbies (3.2 ± 1.1 vs. 3.8 ± 0.8), restoring normal social function (3.4 ± 1.1 vs. 3.9 ± 0.8), and improving sexual satisfaction (2.1 ± 1.2 vs. 3.1 ± 0.8, *p* < 0.0001 for all; Table 6).

**Table 6:** Treatment expectations (goals) and satisfaction towards treatment for anhedonia in MDD

Variables	MDD-ANH (*n* = 282)	MDD non-ANH (*n* = 50)	Physicians (*n* = 60)	*p*-value	
				MDD-ANH vs. Physicians	MDD non-ANH vs. Physicians
Level of importance of treatment goals (scale 1–5) *, **mean (SD)**					
Control depressed mood (e.g., sadness)	3.8 (1.0)	3.7 (1.2)	3.6 (0.8)	0.1445	0.3945
Reduce psychological anxiety (e.g., feeling irritable and worried)	3.9 (1.0)	4.0 (1.2)	3.7 (0.7)	**0.0111**	0.0920
Regain appetite	2.6 (1.1)	2.6 (1.2)	3.6 (0.8)	**< 0.0001**	**< 0.0001**
Improve sleep quality	3.8 (1.0)	3.9 (1.2)	3.7 (0.9)	0.6004	0.3829
Reduce the feeling of fatigue	3.7 (1.0)	3.6 (1.1)	3.6 (0.8)	0.4985	0.8923
Regain interest in hobbies	3.2 (1.1)	3.2 (1.2)	3.8 (0.8)	**< 0.0001**	**0.0081**
Avoid having suicidal thoughts	3.4 (1.2)	3.6 (1.3)	3.9 (0.9)	**0.0006**	0.1641
Improve productivity at work, school, and home (e.g., household chores)	3.3 (1.1)	3.2 (1.1)	3.6 (0.8)	**0.0251**	0.0655
Restore normal social function (i.e., participate and enjoy relationships with family, and friends)	3.4 (1.1)	3.4 (1.2)	3.9 (0.8)	**< 0.0001**	**0.0095**
Have positive emotions (e.g., optimism)	3.7 (1.0)	3.8 (1.1)	3.7 (0.9)	0.9123	0.4712
Regain self-esteem	3.4 (1.0)	3.4 (1.2)	3.6 (0.8)	0.1509	0.3020
Improve sexual satisfaction	2.1 (1.2)	2.4 (1.0)	3.1 (0.8)	**< 0.0001**	**0.0004**
Increase attention span	3.3 (1.0)	3.4 (1.3)	3.4 (0.9)	0.2085	0.7909
**Level of satisfaction with treatment goals (scale 1–9)**^**†**^, **mean (SD)**					
Control depressed mood (e.g., sadness)	4.9 (1.8)	5.1 (2.0)	6.3 (1.4)	**< 0.0001**	**0.0014**
Reduce psychological anxiety (e.g., feeling irritable and worried)	4.7 (1.7)	4.9 (2.2)	6.2 (1.5)	**< 0.0001**	**0.0011**
Regain appetite	5.3 (1.5)	5.6 (1.6)	6.4 (1.3)	**< 0.0001**	**0.0050**
Improve sleep quality	5.1 (2.0)	5.2 (2.0)	6.4 (1.7)	**< 0.0001**	**0.0010**
Reduce the feeling of fatigue	4.7 (1.8)	4.8 (1.9)	5.9 (1.6)	**< 0.0001**	**0.0007**
Regain interest in hobbies	4.7 (1.7)	5.5 (1.7)	5.8 (1.6)	**< 0.0001**	0.4278
Avoid having suicidal thoughts	5.2 (1.7)	5.5 (1.7)	6.0 (1.8)	**0.0019**	0.1521
Improve productivity at work, school, and home (e.g., household chores)	4.9 (1.8)	4.9 (1.8)	5.7 (1.5)	**0.0007**	**0.0186**
Restore normal social function (i.e., participate and enjoy relationships with family, and friends)	4.9 (1.8)	5.2 (1.7)	5.7 (1.6)	**0.0007**	0.1078
Have positive emotions (e.g., optimism)	4.8 (1.7)	5.6 (1.7)	5.9 (1.4)	**< 0.0001**	**0.0223**
Regain self-esteem	4.5 (1.6)	5.0 (1.8)	5.5 (1.6)	**< 0.0001**	0.1336
Improve sexual satisfaction	4.8 (1.6)	5.2 (1.4)	5.1 (1.3)	0.0681	0.8399
Increase attention span	4.6 (1.6)	5.2 (1.6)	5.8 (1.4)	**< 0.0001**	0.0585

ANH, anhedonia; MDD, major depressive disorder; SD, standard deviation

*1-not at all important, 5-extremely important

^†^1-extremely dissatisfied, 9-extremely satisfied

Regarding treatment satisfaction, the top three areas of higher satisfaction for patients with MDD-ANH were regaining appetite (5.3 ± 1.5), avoiding suicidal thoughts (5.2 ± 1.7), and improving sleep quality (5.1 ± 2.0). Patients with MDD non-ANH expressed satisfaction with regaining appetite (5.6 ± 1.6), having positive emotions (5.6 ± 1.7), regaining interest in hobbies (5.5 ± 1.7), and avoiding suicidal thoughts (5.5 ± 1.7). Physicians’ satisfaction levels were notably higher compared to patients, with top-ranking areas including improving sleep quality (6.4 ± 1.7), regaining appetite (6.4 ± 1.3), and controlling depressed mood (6.3 ± 1.4). Physicians also reported significantly higher satisfaction levels than patients with MDD-ANH in pharmaceutical treatments achieving various treatment goals for all factors (*p* < 0.05), except for sexual satisfaction (*p* = 0.0681) (Table 6).

## Discussion

To the best of our knowledge, this is the first cross-sectional, observational study that reported the prevalence of anhedonia among Japanese patients with MDD, along with patient characteristics and experiences along their patient journey. Additionally, this study elucidated physicians’ perspectives towards anhedonia in MDD as well as the expectations and satisfaction levels of both physicians and patients towards pharmaceutical treatments for anhedonia in MDD in Japan.

The prevalence of MDD (PHQ-9 score ≥ 10 and a patient-reported physician diagnosis of depression) was reported as 12.7% among the survey respondents in the present study, with an age- and gender-weighted prevalence of 3.4%, and a notable percentage of respondents experiencing moderate MDD severity. In this study, we used the definition of PHQ-9 score **≥** 10 since this score represents moderate or more in depression severity [30], with a sensitivity of 88% and a specificity of 88% for major depression. Further, PHQ-9 scores **≥** 10 on the Japanese version had a sensitivity of 84% and a specificity of 95% for major depression in Japanese patients [29]. The estimated prevalence of MDD in our study aligns closely with previously reported rates of overall prevalence of 2.0% and lifetime prevalence rate of 5.7% within Japanese population [4, 36], suggesting the consistency and reliability of using self-reported PHQ-9 scores and patient-reported physician diagnoses as valid measures for identifying patients with depression [30, 37]. Furthermore, the prevalence of anhedonia among MDD patients in Japan, as evidenced by the high percentage (66.9%) of patients scoring SHAPS > 2 in the current study is in line with the high prevalence of anhedonia reported in patients with MDD (35–70%) in studies conducted in other geographic regions [10–12]. This suggests that anhedonia may be a consistent feature of MDD across diverse populations highlighting its importance as a key symptom of depression [8], with implications for diagnosis, treatment, and understanding of the overall burden of the disorder.

With regards to physicians’ perspectives on anhedonia in MDD, nine out of ten physicians do not treat anhedonia distinctly, despite agreeing with the definition of anhedonia in MDD. This is noteworthy given that the physicians’ perceived caseload of MDD-ANH patients in the clinical setting was substantially lower than the estimated prevalence of MDD-ANH patients in the current study. Given the high prevalence of MDD-ANH reported in other geographical regions [10–12, 38, 39], this difference suggests a potential underdiagnosis of anhedonia and underestimation of the impact of the disease by physicians in Japan or differences in the terminology of anhedonia between Japanese and English languages. Moreover, physicians demonstrated a moderate level of agreement with statements related to anhedonia (mean scores between 6 and 7), indicating a lack of consensus or clarity in their understanding and management approach toward anhedonia in patients with MDD. This ambiguity has potential clinical implications for MDD patient care quality, especially since anhedonia has been shown to negatively impact patient-related outcomes [1, 27]. Therefore, there is a need to clarify the concept of anhedonia in Japanese language in this field and enhance physicians’ awareness of the impact of anhedonia on the disease burden of MDD and focus efforts on better treatment outreach and management for MDD-ANH patients. This may involve incorporating validated assessment tools for anhedonia, considering adjunctive treatments specifically targeting anhedonia, and providing psychoeducation to patients about the importance of addressing this symptom as part of their treatment plan [8, 27, 40]. 

The patient journey analyzed in the present study from the perspective of patients with MDD-ANH and MDD non-ANH revealed important observations regarding the accessibility of diagnosis and treatment flow. There were no significant differences observed in the duration since depression diagnosis between the two groups. This suggests that patients with MDD-ANH may not be evaluated differently from MDD non-ANH during the diagnostic process. However, it is imperative to note that patients with MDD-ANH scored significantly higher on almost all individual items of the PHQ-9, indicating potentially more severe depression symptoms. Additionally, there was a tendency for a higher percentage of MDD-ANH patients to change treatment due to poor previous treatment results (~ 50%). This raises the inquiry into whether patients with MDD-ANH receive the most effective treatment, at an early or appropriate stage. Further analysis is essential to verify this hypothesis.

In addition, while there were no significant differences in the number of prescriptions or history of previous medication between the two groups, patients with MDD-ANH reported a significantly longer treatment duration with multiple prescriptions compared to MDD non-ANH. This suggests the possibility of more severe depression among patients with MDD-ANH, as evidenced by the higher PHQ-9 scores compared to MDD non-ANH patients. Additionally, this longer treatment duration may indicate that patients with MDD-ANH are more difficult to treat, with inadequate response and residual bothering symptoms. This highlighted the need for further studies to explore this issue. Moreover, patients with MDD-ANH were more likely to switch treatments because they felt the previous treatment was not responding, compared to patients with MDD non-ANH. This finding supports the notion that current treatments may be less effective for patients with MDD-ANH than MDD non-ANH, however future studies are needed for further assessment [8, 27]. Interestingly, despite reporting significantly higher scores on individual items associated with pleasure and cognitive functioning on PHQ-9, patients with MDD-ANH did not differ significantly from MDD non-ANH in terms of the symptoms prompting them to seek medical consultation. This may hint at a possible lack of awareness or motivation to seek support specifically for anhedonia-related symptoms, underscoring the need for improved recognition and understanding of anhedonia among both patients and physicians.

Moreover, the present study reported some discordance between treatment goals and treatment satisfaction among patients with MDD-ANH versus physicians, suggesting a potential implication that current therapeutics may not fully address the key concerns of patients with MDD-ANH. While patients with MDD-ANH prioritize goals related to reducing psychological anxiety, improving sleep quality, and controlling depressed mood, physicians emphasize goals such as restoring normal social function, avoiding suicidal thoughts, and regaining interest in hobbies. Another study on the patient perspective on the effectiveness of esketamine nasal spray in treatment-resistant depression also reported discrepancies on patients’ and clinician’s perspectives about improvement of anhedonia and suicidality; however, the study reported an overall overlapping response from both groups [41]. The treatment goals also differed between MDD non-ANH patients and physicians, where physicians placed more emphasis on restoring function in patients than on the psychological aspects preferred by the MDD non-ANH patients. This underscores the importance of shared decision-making in aligning treatment goals and preferences between patients and physicians [42, 43]. It is worth noting that there is a paucity of studies assessing the treatment preferences of patients and physicians regarding MDD in Japan. Additionally, the level of treatment satisfaction perceived by patients with MDD was lower than that perceived by physicians. This suggests a potential misalignment between physicians and patients in perception of treatment success, irrespective of anhedonia status.

As the original concept of anhedonia has been expanded over the last decades, specifically the neurophysiological basis of anhedonia [44, 45], recent studies highlight the importance of recognizing and treating anhedonia in patients with MDD with specific therapeutic strategies for specific patient subgroups [8, 41]. Existing literature suggests sex-specific neural and biological mechanisms underlying MDD [46–48]. Similarly, research shown that age could affect antidepressant response in patients with MDD [49, 50]. The observation that MDD-ANH group had higher proportions of males and younger patients raise the possibility that these demographic factors may have influenced the differences observed between the anhedonia and non-anhedonia groups such as distinct clinical characteristics, differential treatment expectations and perceived treatment satisfaction. Future research is needed to explore how sex and age impact these outcomes to develop a more personalized and patient-centric approach for managing anhedonia in MDD.

Nevertheless, the present survey findings underscore the clear need for targeted initiatives aimed at enhancing physician awareness and refining treatment outreach and management for MDD-ANH patients. Additionally, efforts to enhance awareness and motivation for seeking support for anhedonia-related symptoms among both patients and physicians are crucial for optimizing patient outcomes within this population. Moreover, addressing the disparities in treatment expectations and satisfaction between patients and physicians warrants further research.

The present study also has several limitations. One notable limitation is the relatively small sample size of MDD non-ANH patients, which may potentially impact the statistical power of the study with a lack of statistical differences between MDD-ANH and MDD non-ANH patients. Secondly, given that the survey utilized an online questionnaire, certain segments of the population may have been underrepresented. Specifically, respondents lacking internet access or familiarity with online platforms, as well as those who are institutionalized, elderly, or experiencing severe comorbidities or disabilities, may not have been adequately represented in the study sample. Thirdly, due to the self-reported nature of the study, verification of the responses could not be performed, and causal inference is limited by the potential for recollection bias despite efforts made to minimize intentional false reporting by participants. Additionally, this study relied on the physicians’ estimation and perception of anhedonia among their MDD patients. While this provided valuable insights into how physicians perceived anhedonia in MDD patients within a real-world clinical setting, this approach may introduce subjectivity and potential variability. Future studies may be needed to validate this study’s findings in a clinical setting where the physician diagnosis of MDD could be validated. Furthermore, inherent to observational studies, there exists a potential for selection bias, as respondents may not fully represent the broader population of patients diagnosed with MDD in Japan.

## Conclusions

This is the first study to report the prevalence of MDD with anhedonia in Japan. The prevalence of anhedonia among MDD patients was high. Additionally, findings from this study highlight a perceived lower prevalence of anhedonia and a potential underestimation of the impact of anhedonia on MDD patients among physicians in Japan. Moreover, the study suggests that MDD-ANH patients may have challenges accessing appropriate treatment, as they may be less likely to seek support despite experiencing greater symptom severity than MDD non-ANH patients. This underscores the need to enhance physicians’ awareness of the impact of anhedonia to drive better treatment outreach that distinctly addresses the needs of MDD-ANH patients. Furthermore, while similarities in treatment expectations were observed among MDD patients (regardless of anhedonia status), there was discordance between physicians and MDD patients in treatment expectations and satisfaction especially those with anhedonia. This suggests the importance of aligning patient and physician treatment expectations to enhance patient-physician alliance for improved treatment outcomes, patient satisfaction, and quality of care, thereby reducing the disease burden associated with MDD and anhedonia.

## Electronic supplementary material

Below is the link to the electronic supplementary material.

Supplementary Material 1

## Data Availability

No datasets were generated or analysed during the current study.

## Abbreviations

ANH: anhedonia
BMI: body mass index
CCI: Charlson Comorbidity Index
DALYs: disability-adjusted life years
DSM-5-RT: The Diagnostic and Statistical Manual of Mental Disorders, Revised Text
GEP: Good Epidemiological Practices
ISPOR: International Society for Pharmacoeconomics and Outcomes Research
MDB: Medical Database Provision Business (Japan)
MDD: major depressive disorder
MDD-ANH: MDD patients with anhedonia
MDD non-ANH: MDD patients without anhedonia
PHQ-9: Patient Health Questionnaire-9
SD: standard deviation
SHAPS: Snaith-Hamilton Pleasure Scale

## References

[1] Cheng C, Herr K, Jeon HJ, Kato T, Ng CH, Yang YK. A Delphi consensus on clinical features, diagnosis and treatment of major depressive disorder patients with anhedonia amongst psychiatrists in the Asia-Pacific. Front Psychiatry. 2024;15. 10.3389/fpsyt.2024.1338063 PMC10920342 38463427

[2] Santomauro DF, Mantilla Herrera AM, Shadid J, Zheng P, Ashbaugh C, Pigott DM. Global prevalence and burden of depressive and anxiety disorders in 204 countries and territories in 2020 due to the COVID-19 pandemic. Lancet. 2021;398:1700–12. 34634250 10.1016/S0140-6736(21)02143-7 PMC8500697

[3] Vos T, Lim SS, Abbafati C, Abbas KM, Abbasi M, Abbasifard M. Global burden of 369 diseases and injuries in 204 countries and territories, 1990–2019: a systematic analysis for the global burden of disease study 2019. Lancet. 2020;396:1204–22. 33069326 10.1016/S0140-6736(20)30925-9 PMC7567026

[4] Ishikawa H, Tachimori H, Takeshima T, Umeda M, Miyamoto K, Shimoda H. Prevalence, treatment, and the correlates of common mental disorders in the mid 2010′s in japan: the results of the world mental health Japan 2nd survey. J Affect Disord. 2018;241:554–62. 30153639 10.1016/j.jad.2018.08.050

[5] Nakao M, Takeuchi T, Yoshimasu K. A proposed approach to suicide prevention in japan: the use of self-perceived symptoms as indicators of depression and suicidal ideation. Environ Health Prev Med. 2008;13:313–21. 19568891 10.1007/s12199-008-0048-7 PMC2698228

[6] Guo L. Anhedonia - what is it, causes, diagnosis, and more. Osmosis by Elsevier. https://www.osmosis.org/answers/anhedonia#::text = Anhedonia refers to the loss,to as the ventral striatum.

[7] Trøstheim M, Eikemo M, Meir R, Hansen I, Paul E, Kroll SL. Assessment of anhedonia in adults with and without mental illness a systematic review and meta-analysis. JAMA Netw Open. 2020;3:e2013233. 32789515 10.1001/jamanetworkopen.2020.13233 PMC7116156

[8] Serretti A. Anhedonia and depressive disorders. Clin Psychopharmacol Neurosci. 2023;21:401–9. 37424409 10.9758/cpn.23.1086 PMC10335915

[9] American Psychiatric Association. Diagnostic and statistical manual of mental disorders, text revision (DSM-5-TR). In: text revision. 2022.

[10] Cao B, Zhu J, Zuckerman H, Rosenblat JD, Brietzke E, Pan Z. Pharmacological interventions targeting anhedonia in patients with major depressive disorder: A systematic review. Prog Neuropsychopharmacol Biol Psychiatry. 2019;92:109–17. 30611836 10.1016/j.pnpbp.2019.01.002

[11] Shankman SA, Katz AC, DeLizza AA, Sarapas C, Gorka SM, Campbell ML. The different facets of anhedonia and their associations with different psychopathologies. Anhedonia: A comprehensive handbook. Dordrecht: Springer Netherlands; 2014. pp. 3–22.

[12] Pelizza L, Ferrari A. Anhedonia in schizophrenia and major depression: state or trait? Ann Gen Psychiatry. 2009;8:22. 19811665 10.1186/1744-859X-8-22 PMC2764701

[13] Mazza M, Squillacioti MR, Pecora RD, Janiri L, Bria P. Effect of Aripiprazole on self-reported anhedonia in bipolar depressed patients. Psychiatry Res. 2009;165:193–6. 18973955 10.1016/j.psychres.2008.05.003

[14] Liang S, Wu Y, Hanxiaoran L, Greenshaw AJ, Li T. Anhedonia in depression and schizophrenia: brain reward and aversion circuits. Neuropsychiatr Dis Treat. 2022;18:1385–96. 35836582 10.2147/NDT.S367839 PMC9273831

[15] Christensen MC, Ren H, Fagiolini A. Emotional blunting in patients with depression. Part II: relationship with functioning, well-being, and quality of life. Ann Gen Psychiatry. 2022;21. 10.1186/s12991-022-00392-4 PMC9210577 35725552

[16] Fusar-Poli P, Estradé A, Stanghellini G, Esposito CM, Rosfort R, Mancini M. The lived experience of depression: a bottom‐up review co‐written by experts by experience and academics. World Psychiatry. 2023;22:352–65. 37713566 10.1002/wps.21111 PMC10503922

[17] Wang S, Leri F, Rizvi SJ. Anhedonia as a central factor in depression: neural mechanisms revealed from preclinical to clinical evidence. Prog Neuropsychopharmacol Biol Psychiatry. 2021;110. 10.1016/j.pnpbp.2021.110289 33631251

[18] Watanabe K, Fujimoto S, Marumoto T, Kitagawa T, Ishida K, Nakajima T. Therapeutic potential of Vortioxetine for anhedonia-like symptoms in depression: a post hoc analysis of data from a clinical trial conducted in Japan. Neuropsychiatr Dis Treat. 2022;18:363–73. 35221687 10.2147/NDT.S340281 PMC8865902

[19] Progress in Mind. Psychiatry and Neurology Resource Centre. Why should we care about anhedonia in major depressive disorder?.

[20] Lally N, Nugent AC, Luckenbaugh DA, Niciu MJ, Roiser JP, Zarate CA. Neural correlates of change in major depressive disorder anhedonia following open-label ketamine. J Psychopharmacol. 2015;29:596–607. 25691504 10.1177/0269881114568041 PMC5116382

[21] Lindahl J, Asp M, Ståhl D, Tjernberg J, Eklund M, Björkstrand J. Add-on Pramipexole for anhedonic depression: study protocol for a randomised controlled trial and open-label follow-up in lund, Sweden. BMJ Open. 2023;13:e076900. 38035737 10.1136/bmjopen-2023-076900 PMC10689415

[22] Fujiwara S, Kimura F, Hosokawa T, Ishida S, Sugino M, Hanafusa T. Anhedonia in Japanese patients with parkinson’s disease. Geriatr Gerontol Int. 2011;11:275–81. 21241445 10.1111/j.1447-0594.2010.00678.x

[23] Miura S, Kida H, Nakajima J, Noda K, Nagasato K, Ayabe M. Anhedonia in Japanese patients with parkinson’s disease: analysis using the Snaith–Hamilton pleasure scale. Clin Neurol Neurosurg. 2012;114:352–5. 22137783 10.1016/j.clineuro.2011.11.008

[24] Harada T, Ishizaki F, Horie N, Katsuoka H, Nitta Y, Yamada T. Clinical characteristics of anhedonia in Japanese patients with parkinson’s disease. Int Med J. 2011;18.

[25] Kaji Y, Hirata K. Apathy and anhedonia in parkinson’s disease. ISRN Neurol. 2011;2011:1–9. 10.5402/2011/219427 PMC3263557 22389809

[26] Takehara K, Tachibana Y, Yoshida K, Mori R, Kakee N, Kubo T. Prevalence trends of pre- and postnatal depression in Japanese women: A population-based longitudinal study. J Affect Disord. 2018;225:389–94. 28846961 10.1016/j.jad.2017.08.008

[27] Kuntz L. Anhedonia in major depressive disorder: Understanding patient burden. Psychiatric Times. 2023.

[28] Khazanov GK, Forbes CN, Dunn BD, Thase ME. Addressing anhedonia to increase depression treatment engagement. Br J Clin Psychol. 2022;61:255–80. 34625993 10.1111/bjc.12335

[29] Muramatsu K, Kamijima K, Yoshida M, Otsubo T, Miyaoka H, Muramatsu Y. The patient health questionnaire, Japanese version: validity according to the mini-international neuropsychiatry interview-plus. Psychol Rep. 2007;I:952–60. 10.2466/pr0.101.3.952-960 18232454

[30] Kroenke K, Spitzer RL, Williams JBW. The PHQ-9: validity of a brief depression severity measure. J Gen Intern Med. 2001;16:606–13. 11556941 10.1046/j.1525-1497.2001.016009606.x PMC1495268

[31] Nagayama H, Kubo S, Hatano T, Hamada S, Maeda T, Hasegawa T, et al. Validity and reliability assessment of a Japanese version of the Snaith-Hamilton pleasure scale. Intern Med. 2012;51:865–9. 22504240 10.2169/internalmedicine.51.6718

[32] Leventhal AM, Unger JB, Audrain-McGovern J, Sussman S, Volk HE, Strong DR. Measuring anhedonia in adolescents: A psychometric analysis. J Pers Assess. 2015;97:506–14. 25893676 10.1080/00223891.2015.1029072 PMC4545400

[33] Snaith RP, Hamilton M, Morley S, Humayan A, Hargreaves D, Trigwell P. A scale for the assessment of hedonic tone the Snaith–Hamilton pleasure scale. Br J Psychiatry. 1995;167:99–103. 7551619 10.1192/bjp.167.1.99

[34] United Nations. World Population Prospects 2022. 2022.

[35] Charlson ME, Pompei P, Ales KL, MacKenzie CR. A new method of classifying prognostic comorbidity in longitudinal studies: development and validation. J Chronic Dis. 1987;40:373–83. 3558716 10.1016/0021-9681(87)90171-8

[36] Demiya S, Takeno S, Kim S, Lee W, Sakai Y. EPH14 prevalence of depression in Japan and the US populations before and during the COVID-19 pandemic: A retrospective observational study using real-world data. Value Health. 2022;25:S193.

[37] Sun Y, Fu Z, Bo Q, Mao Z, Ma X, Wang C. The reliability and validity of PHQ-9 in patients with major depressive disorder in psychiatric hospital. BMC Psychiatry. 2020;20. 10.1186/s12888-020-02885-6 PMC7525967 32993604

[38] Franken IHA, Rassin E, Muris P. The assessment of anhedonia in clinical and non-clinical populations: further validation of the Snaith–Hamilton pleasure scale (SHAPS). J Affect Disord. 2007;99:83–9. 16996138 10.1016/j.jad.2006.08.020

[39] Buckner JD, Joiner TE, Pettit JW, Lewinsohn PM, Schmidt NB. Implications of the dsm’s emphasis on sadness and anhedonia in major depressive disorder. Psychiatry Res. 2008;159:25–30. 18334272 10.1016/j.psychres.2007.05.010 PMC3688280

[40] Su Y-A, Si T. Progress and challenges in research of the mechanisms of anhedonia in major depressive disorder. Gen Psychiatr. 2022;35:e100724. 35309242 10.1136/gpsych-2021-100724 PMC8883269

[41] Pepe M, Bartolucci G, Marcelli I, Pesaresi F, Brugnami A, Caso R. The patient’s perspective on the effects of intranasal Esketamine in treatment-resistant depression. Brain Sci. 2023;13. 10.3390/brainsci13101494 PMC10604956 37891860

[42] Christensen MC, Wong CMJ, Baune BT. Symptoms of major depressive disorder and their impact on psychosocial functioning in the different phases of the disease: do the perspectives of patients and healthcare providers differ? Front Psychiatry. 2020;11. 10.3389/fpsyt.2020.00280 PMC7193105 32390877

[43] Menear M, Girard A, Dugas M, Gervais M, Gilbert M, Gagnon M-P. Personalized care planning and shared decision making in collaborative care programs for depression and anxiety disorders: A systematic review. PLoS ONE. 2022;17:0268649. 10.1371/journal.pone.0268649 PMC9187074 35687610

[44] Camardese G, Risio L, Nicola M, Pucci L, Cocciolillo F, Bria P. Changes of dopamine transporter availability in depressed patients with and without anhedonia: A 123 I-N-ω-Fluoropropyl-Carbomethoxy-3β- (4-Iodophenyl)tropane SPECT study. Neuropsychobiology. 2014;70:235–43. 25613182 10.1159/000368117

[45] D’Onofrio AM, Pizzuto DA, Batir R, Perrone E, Cocciolillo F, Cavallo F. Dopaminergic dysfunction in the left putamen of patients with major depressive disorder. J Affect Disord. 2024;357:107–15. 38636713 10.1016/j.jad.2024.04.044

[46] Vetter JS, Spiller TR, Cathomas F, Robinaugh D, Brühl A, Boeker H, et al. Sex differences in depressive symptoms and their networks in a treatment-seeking population– a cross-sectional study. J Affect Disord. 2021;278:357–64. 33002727 10.1016/j.jad.2020.08.074 PMC8086368

[47] LeGates TA, Kvarta MD, Thompson SM. Sex differences in antidepressant efficacy. Neuropsychopharmacol. 2019;44:140–54. 10.1038/s41386-018-0156-z PMC6235879 30082889

[48] Labaka A, Goñi-Balentziaga O, Lebeña A, Pérez-Tejada J. Biological sex differences in depression: A systematic review. Biol Res Nurs. 2018;20:383–92. 29759000 10.1177/1099800418776082

[49] Zheng W, Gu L, Tan J, Zhou Y, Wang C, Lan X et al. Comparison of the antianhedonic effects of Repeated-dose intravenous ketamine in older and younger adults with major depressive episode. Curr Neuropharmacol 23:232–9. 10.2174/1570159X23666240923112548 PMC11793042 39318021

[50] Strawn JR, Mills JA, Suresh V, Mayes T, Gentry MT, Trivedi M, et al. The impact of age on antidepressant response: A mega-analysis of individuals with major depressive disorder. J Psychiatr Res. 2023;159:266–73. 36774767 10.1016/j.jpsychires.2023.01.043 PMC9993423

