# Supplementary material for: Understanding anhedonia in major depressive disorder in Japan: epidemiology and unmet needs from patients’ and physicians’ perspectives
Source: BMC Psychiatry. 2025 Jul 1;25:631. doi: 10.1186/s12888-025-07089-4 (PMC12219931; doi:10.1186/s12888-025-07089-4)
Supplement: Supplementary file 1 — Supplementary Material 1 [file 12888_2025_7089_MOESM1_ESM.docx]

**SUPPLEMENTARY MATERIALS**

**Table S1.** Patients’ characteristics (unweighted)

| **Variables** | **MDD-ANH**  **(n=307)** | **MDD non-ANH**  **(n=54)** |
| --- | --- | --- |
|  |  |  |
| **Sociodemographic characteristics** | | |
| **Sex, n (%)** | | |
| Male | 202 (65.8) | 25 (46.3) |
| Female | 105 (34.2) | 29 (53.7) |
| **Age, mean (SD)** | 48.5 (10.5) | 50.3 (9.0) |
| **Age distribution, n (%)** | | |
| 18 to <25 | 3 (1.0) | 0 (0.0) |
| 25 to <35 | 29 (9.5) | 2 (3.7) |
| 35 to <45 | 77 (25.1) | 9 (16.7) |
| 45 to <55 | 109 (35.5) | 26 (48.2) |
| 55 to <65 | 70 (22.8) | 14 (25.9) |
| 65 and older | 19 (6.2) | 3 (5.6) |
| **Education, n (%)** | | |
| Elementary school | 0 (0.0) | 0 (0.0) |
| Junior high school | 8 (2.6) | 1 (1.9) |
| High school | 88 (28.7) | 16 (29.6) |
| 2-year college | 42 (13.7) | 7 (13.0) |
| 4-year college | 129 (42.0) | 19 (35.2) |
| Graduate school or above | 27 (8.8) | 8 (14.8) |
| No school | 0 (0.0) | 0 (0.0) |
| Others | 11 (3.6) | 3 (5.6) |
| Decline to answer | 2 (0.7) | 0 (0.0) |
| **Employment status, n (%)** | | |
| Employed* | 212 (69.1) | 39 (72.2) |
| Non-employed^†^ | 100 (32.6) | 16 (29.6) |
| **General health characteristics** | | |
| **BMI, mean (SD)** | 23.8 (5.4) | 22.6 (4.1) |
| **BMI categories, n (%)** | | |
| Underweight (<18.5) | 36 (11.7) | 8 (14.8) |
| Normal weight (18.5 to <25) | 170 (55.4) | 35 (64.8) |
| Overweight (25 to <30) | 67 (21.8) | 8 (14.8) |
| Obese (30 or greater) | 34 (11.1) | 3 (5.6) |
| Unknown | 0 (0.0) | 0 (0.0) |
| **Frequency of smoking, n (%)** | | |
| Every day | 96 (31.3) | 12 (22.2) |
| Some days | 25 (8.1) | 1 (1.9) |
| Not at all | 186 (60.6) | 41 (75.9) |
| **Frequency of consuming alcohol, n (%)** | | |
| Every day | 83 (27.0) | 13 (24.1) |
| Some days | 115 (37.5) | 21 (38.9) |
| Not at all | 109 (35.5) | 20 (37.0) |
| **CCI, mean (SD)** | 1.1 (1.4) | 1.4 (1.7) |
| **CCI score categories, n (%)** | | |
| 0 | 130 (42.4) | 20 (37.0) |
| 1 | 83 (27.0) | 19 (35.2) |
| 2 | 52 (16.9) | 5 (9.3) |
| 3+ | 42 (13.7) | 10 (18.5) |
| ANH, anhedonia; BMI, body mass index; CCI, Charlson Comorbidity Index; MDD, major depressive disorder; SD, standard deviation.  *Employed comprises of respondents who are employed full-time or part-time and self-employed.  ^†^Non-employed comprises of respondents who are homemakers, retired, student, long-term disability (long-term leave of absence due to illness of your own [more than 3 months]), short-term disability (short-term leave of absence due to illness of your own [less than 3 months]), not employed but looking for work, not employed and not looking for work. | | |
